# A novel anticancer agent SNG1153 inhibits growth of lung cancer stem/progenitor cells

**DOI:** 10.18632/oncotarget.9783

**Published:** 2016-06-02

**Authors:** Shiyang Liu, Yuming Guo, Jing Wang, Hai Zhu, Yuqing Han, Mingji Jin, Jun Wang, Congya Zhou, Junfeng Ma, Qingcong Lin, Zhaoyi Wang, Kun Meng, Xueqi Fu

**Affiliations:** ^1^ School of Life Sciences, Jilin University, Changchun, P.R. China; ^2^ Beijing Shenogen Biomedical Co., Ltd, Beijing, P.R. China

**Keywords:** SNG1153, lung cancer, CSC, tumorsphere, β-catenin

## Abstract

Lung cancer is the leading cause of cancer-related death in both men and women. Lung cancer contains a small population of cancer cells with stem-like features known as cancer stem cells (CSCs). CSCs are often more resistant to current therapeutic treatments. Thus, it is urgent to develop a novel agent that is able to inhibit CSCs growth. In this study, we examined the ability of SNG1153, a novel chemical agent to inhibit the growth of lung CSCs. We found that SNG1153 inhibited growth and induced apoptosis in established lung cancer cells. We also found that SNG1153 inhibited the tumorsphere formation and decreased CD133-positive (lung CSC marker) cancer cells. SNG1153 was able to attenuate tumor formation in NOD/SCID (non-obese diabetic/severe combined immunodeficient) mice injected with lung tumorsphere cells. We further demonstrated that SNG1153 induced β-catenin phosphorylation and down-regulated β-catenin. Our results thus demonstrate that SNG1153 effectively inhibits the growth of lung CSCs and suggest that SNG1153 may be a novel therapeutic agent to treat human lung cancer.

## INTRODUCTION

Human lung cancer is the most common cause of cancer-related mortality in the world and affects more than one million people yearly [1, 2]. Despite the continuous efforts to improve the therapeutic response, the overall five-year survival rate is still very low [1].

Recently, it has been demonstrated that a subpopulation of cancer cells harboring stem cell features known as cancer stem cells sustain the growth of tumors and are often resistant to the current cancer therapies. CSCs are a small subpopulation of undifferentiated cells within tumors that are responsible for tumor initiation, maintenance, and metastasis [3–5]. CSCs have been identified in various human tumors, such as breast, brain, prostate, pancreatic, colon and lung cancers [1, 3, 6–11]. CSCs-enriched cancer cell populations form tumorsphere in low-adherence culture *in vitro* [12, 13] and exhibit the ability to form tumors at limiting dilutions *in vivo* [1]. Distinct markers have been identified for purification of cancer stem cells, such as CD133, CD44^high^/CD24^low^, ABCG2, ALDH-1 [1, 6, 7, 9, 11, 14–17].

The current cancer therapies usually lack efficacy in long-term outcome because they fail to target CSCs [18]. Thus, developing new therapeutics targeting CSCs is opening up a new avenue for drug discovery [19, 20]. Aberrant stem cell signaling pathways (such as WNT, FGF, Notch, Hedgehog, and TGF/BMP and so on) result in the transformation of normal stem cells to cancer stem cells, and induce various diseases, including cancer, fibrosis and degenerative diseases [21–27]. Among them, WNT is one of the most important signaling pathways in the drug discovery field over the past decade and has been reported to maintain CSCs of myeloid leukemia, melanoma, breast, colon, liver, and lung cancers [17, 28]. The most advanced clinical compound, salinomycin was reported to inhibit mammary tumor growth *in vivo* and induce increased epithelial differentiation of tumor cells [29]. A subsequent study has demonstrated that salinomycin exerts anti-CSC effects by inhibiting WNT signaling cascade through interfering with LPR6 phosphorylation [30]. Here we show that SNG1153 induces β-catenin phosphorylation and then down-regulates β-catenin, a crucial component of the WNT pathway, which plays a key role in cancer stem cells.

SNG1153 is a synthetic compound derived from icaritin which is purified from *Epimedium Genus*, a traditional Chinese herbal medicine. Icaritin exhibits multiple biological activities, such as cardiovascular function improvement, hormone regulation, modulation of immunological function and antitumor activity [31–38].

In this study, we investigated the effects of SNG1153 on the growth of the stem/progenitor cells derived from lung cancer H460 cells.

## RESULTS

### SNG1153 exhibits growth inhibitory activity in lung cancer cells

**Figure 1:**
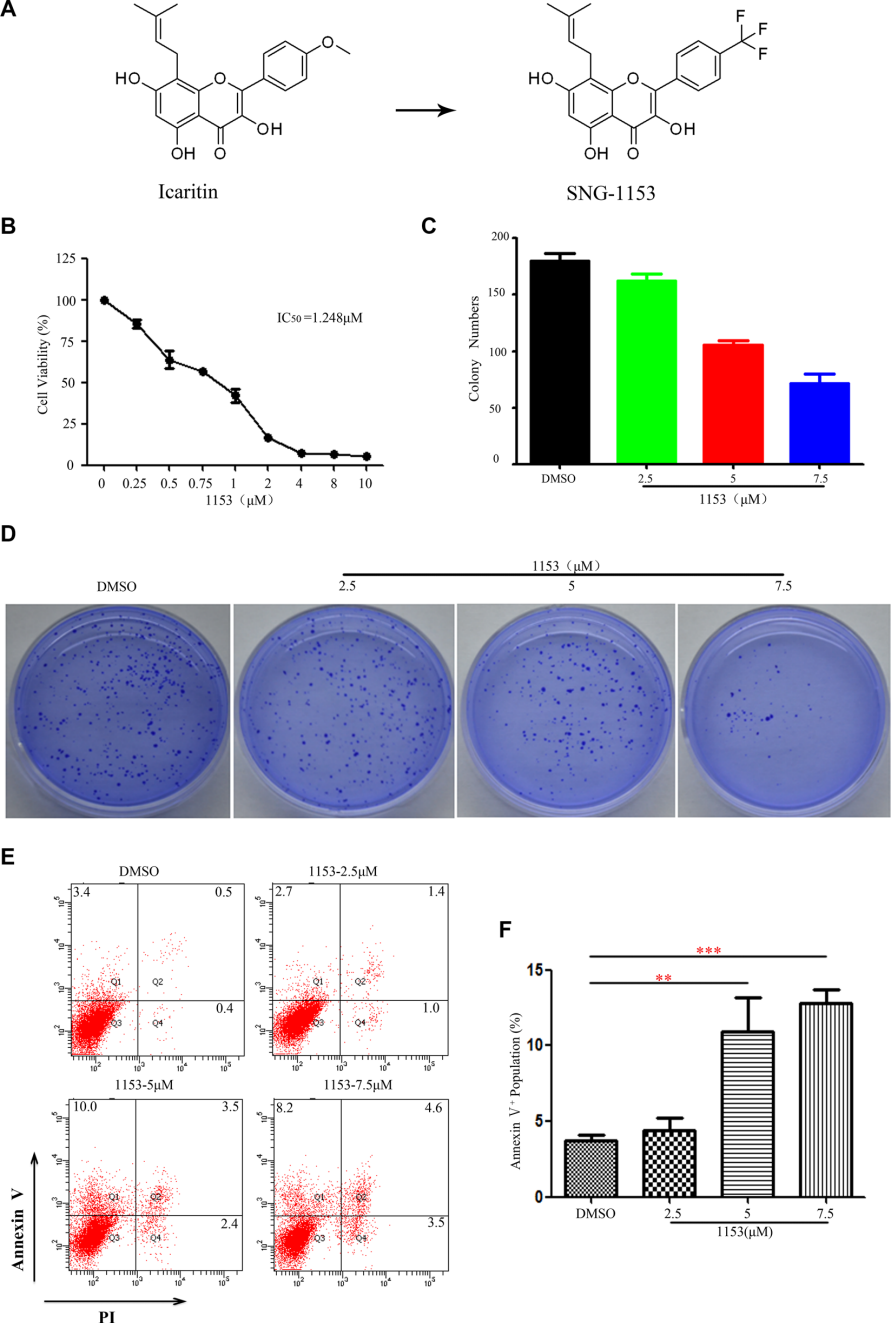
SNG1153 inhibits growth and induces apoptosis in H460 lung cancer cells (**A**) Chemical structure of icaritin and SNG1153. (**B**) SNG1153 inhibited H460 cell growth. H460 cells were treated with the indicated concentrations of SNG1153 for 48 h and cell growth was measured by the CCK8 assay. Point, mean (*n* = 3); bars, SD. (**C** and **D**) Clonogenic assay of H460 cells treated with SNG1153. The cells were pretreated with SNG1153 or DMSO for 24 h then seeded in medium containing 0.3% soft agar in the upper layer and 0.6% soft agar in the lower layer. After 10 days, the cell colonies were counted under a microscope (**E** and **F**) SNG1153 induced cell apoptosis. H460 cells were treated with the indicated concentrations of SNG1153 for 48 h and collected for apoptosis assay. The results represent three independent experiments. Points, mean (*n* = 3); bars, SEM. 1153: SNG1153.

SNG1153 is a derivate of icaritin, a hydrolytic product of icariin from *Epimedium* (Figure 1A). Icaritin has many pharmacological and biological activities, such as the treatment of liver cancer, breast cancer and other diseases [31–38]. To evaluate the effects of SNG1153 on the growth of lung cancer cells, CCK8 assay was performed in the established lung cancer H460 cells treated with various concentrations of SNG1153 for 48 h. We found that SNG1153 effectively inhibited the growth of H460 cells in a dose-dependent manner (Figure 1B). Taxol and salinomycin were used as controls (Figure S1A, S1B). Additionally, SNG1153 inhibited the colony forming activity of H460 cells in a dose-dependent fashion (Figure 1C, 1D). These data suggested that SNG1153 exerts potent inhibitory effects on lung cancer cell growth *in vitro*. SNG1153 exhibits a better bioavailability after orally administration and the IC_50_ of SNG1153 is only one sixth of icaritin in H460 cells (Figure S2). These results indicated SNG1153 has better bioavailability and safety than icaritin.

During the experiments, we noticed that there were floating cells when H460 cells treated with SNG1153. We then decided to determine whether SNG1153 induced cell apoptosis. H460 cells were treated with different concentrations of SNG1153 for 48 h, stained with Annexin V and PI and analyzed with flow cytometry to examine the early stage apoptotic cells (annexin V^+^/PI^−^), the late stage apoptosis cells or necrotic cells (annexinV^+^/PI^+^), and cell debris (annexinV^−^/PI^+^). We found that SNG1153 indeed induced apoptosis in H460 cells (Figure 1E, 1F).

### SNG1153 inhibits the growth of tumorsphere cells derived from lung cancer H460 cells

**Figure 2:**
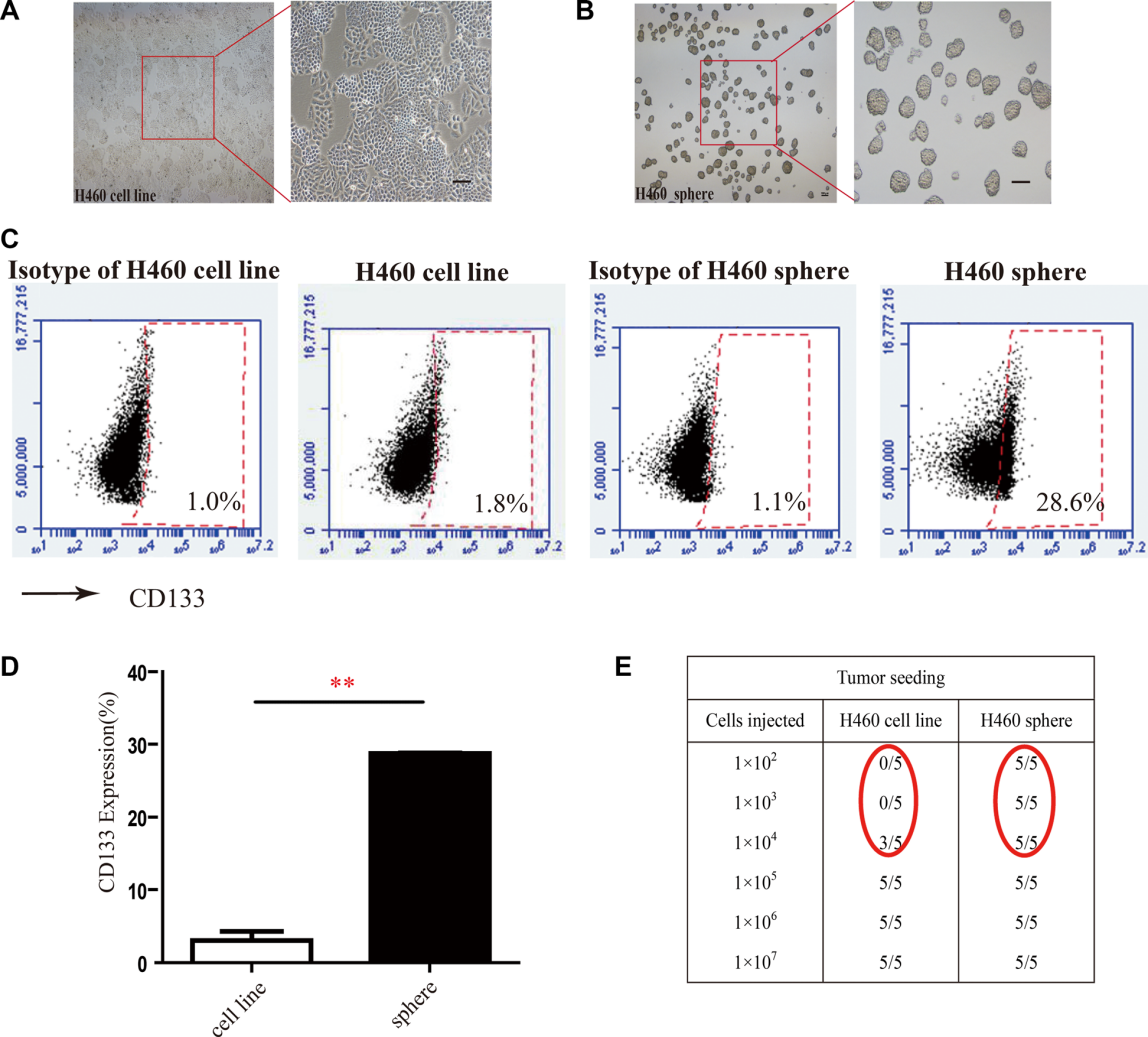
Tumorsphere formation enriches cancer stem-like cells from H460 cells (**A** and **B**) Morphology of H460 cells and tumorspheres from lung cancer H460 cells Scale bar 100 μm. (**C** and **D**) CD133 positive populations in H460 adherent cells and tumorsphere cells were compared. H460 cells were cultured for 5 days in tumorsphere medium or cultured for 2 days in 10% FBS-1640 medium, subsequently the 7-AAD negative cells (living cells) were stained with anti-CD133 antibody and analyzed with flow cytometry. Points, mean (*n* = 3); bars, SEM. (**P < 0.01); (**E**) Tumor formation in NOD/SCID mice xenografted with different number of H460 adherent cells or H460 tumorsphere cells.

As cancer stem/progenitor cells are refractory to most chemotherapy agents [4, 39, 40], we next decided to examine whether SNG1153 affects growth of lung CSCs. It has been reported that tumorspheres are capable of yielding secondary tumorspheres and differentiating along multiple lineages [12]. Our results demonstrated H460 cells in tumorsphere culture medium for 7 days were able to form tumorspheres (Figure 2A, 2B).

CD133 has been identified as a stem-like cell marker and used to enrich H460 lung cancer stem cells [1, 41, 42]. We examined the population of cells with CD133 positive phenotype in tumorsphere cells derived from H460 cells and found that CD133 positive cell population was 15-fold higher in H460 tumorsphere compared to H460 adherent cells (Figure 2C, 2D). We further found that as low as 1 × 10^2^ tumorsphere cells were capable of forming tumors in NOD/SCID mice while it required 1 × 10^4^ H460 adherent cells to form tumors in NOD/SCID mice (Figure 2E). These results indicated that tumorspheres highly enrich cancer stem/progenitor cells.

**Figure 3:**
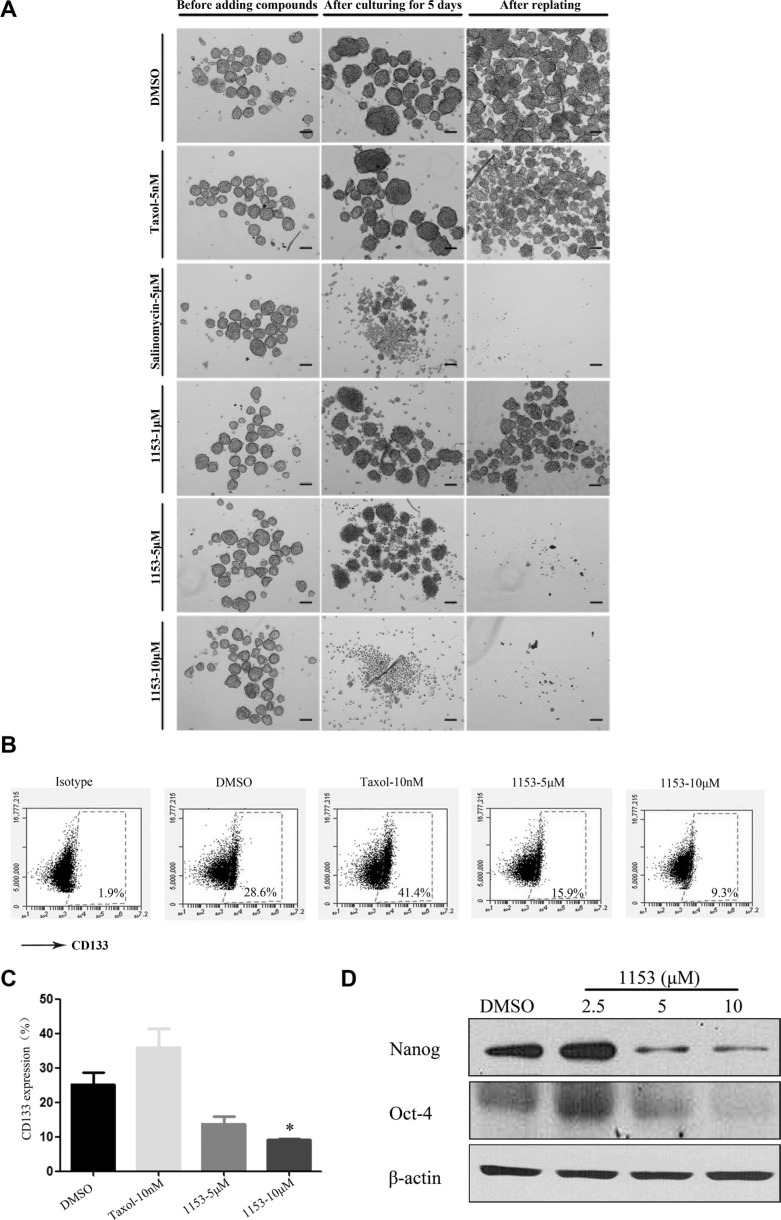
SNG1153 inhibits growth of H460 tumorsphere (**A**) H460 tumorsphere cells were treated with the indicated concentrations of SNG1153 for 5 days. Taxol was used as a negative control and salinomycin as a positive control. After 5 days, tumospheres were photographed and re-seeded for second generation of tumorsphere in the absence of compounds for another 5 days to measure the self-renewal ability. Scale bar 100 μm. (**B** and **C**) H460 tumorsphere cells were treated with the indicated concentrations of DMSO, SNG1153 or Taxol for 5 days and then the 7-AAD negative cells were analyzed with flow cytometric analysis after anti-CD133 antibody staining. Points, mean (*n* = 3); bars, SEM. (*P < 0.05 versus Control group). (**D**) Western blot analysis of Nanog and Oct-4 expression in H460 tumorsphere cells treated with SNG1153. All data are representative results from three independent experiments. 1153:SNG1153.

To examine the effect of SNG1153 on tumorsphere formation *in vitro*, we exposed tumorsphere cells derived from H460 cells to different concentrations of SNG1153 for 5 days and then cultured one additional passage in the absence of SNG1153. The chemotherapy agent taxol and anti-CSCs agent salinomycin were also included [29, 43]. Our data showed that both SNG1153 and salinomycin decreased the size of tumorspheres compared to vehicle controls. In contrast, taxol increased tumorsphere size significantly (Figure 3A). We next sought to examine the effects of SNG1153 on CSC self-renewal ability. The first generation tumorspheres were dissociated and passed for the second generation of tumorspheres. Both salinomycin and SNG1153 suppressed the self-renewal of lung cancer stem cells derived from H460 cells while the vehicle and taxol had no effect (Figure 3A).

Furthermore, SNG1153 decreased the CD133 positive population of H460 tumorsphere with a dose-dependent manner. In contrast, taxol treatment increased the CD133 positive cell population by 2-fold (Figure 3B, 3C).

It has been reported that stemness genes such as Oct4 (octamer-binding transcription factor 4)/Nanog promote lung tumor malignancy and metastasis, by inducing cancer stem cell-like properties [44]. We further investigated the effect of SNG1153 on the Oct4/Nanog expression. We found that SNG1153 treatment significantly reduced the gene expression of the Oct4/Nanog in tumorsphere cells (Figure 3D). These results indicated that SNG1153 inhibits the growth of tumorsphere cells derived from lung cancer H460 cells.

### SNG1153 reduces the tumor formation ability of tumorsphere cells *in vivo*

**Figure 4:**
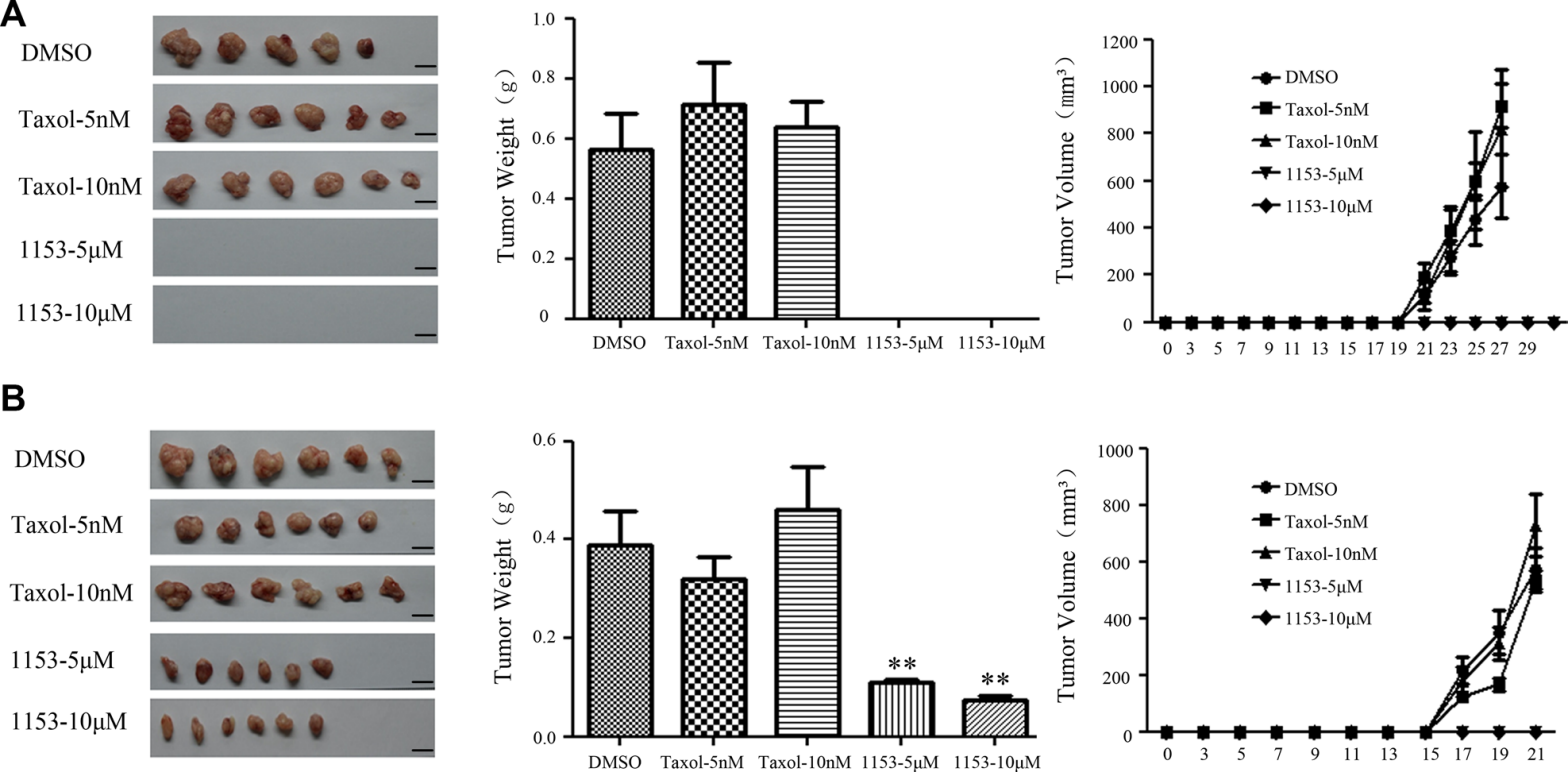
SNG1153 attenuates tumor formation of H460 tumorsphere cells *in vivo* H460 tumorsphere cells treated with SNG1153 or Taxol for 2 days were inoculated in NOD/SCID mice. 5 × 10^2^ (**A**) or 5 × 10^4^ (**B**) of 7-AAD negative cells was used. Tumor volume weights were measured every other day for one month. Scale bar 1 cm. Points, mean (*n* = 6), bars, SEM. (**P < 0.01versus Control group). 1153: SNG1153.

We next want to assess the *in vivo* tumor-seeding ability of tumorsphere cells treated with SNG1153. We observed that SNG1153 pretreatment resulted in a reduction of the ability of tumorsphere cells to form tumors *in vivo.* The sites inoculated with 5 × 10^2^ cells survived from SNG1153 pretreatment failed to form tumors while the vehicle and taxol pretreatment had no effect (Figure 4A, Table S1). The same results were obtained in the 5 × 10^4^ groups (Figure 4B). Taken together, these results indicated that SNG1153 effectively inhibits the growth of lung cancer stem/progenitor cells.

### SNG1153 suppresses β-catenin protein level in tumorsphere cells

**Figure 5:**
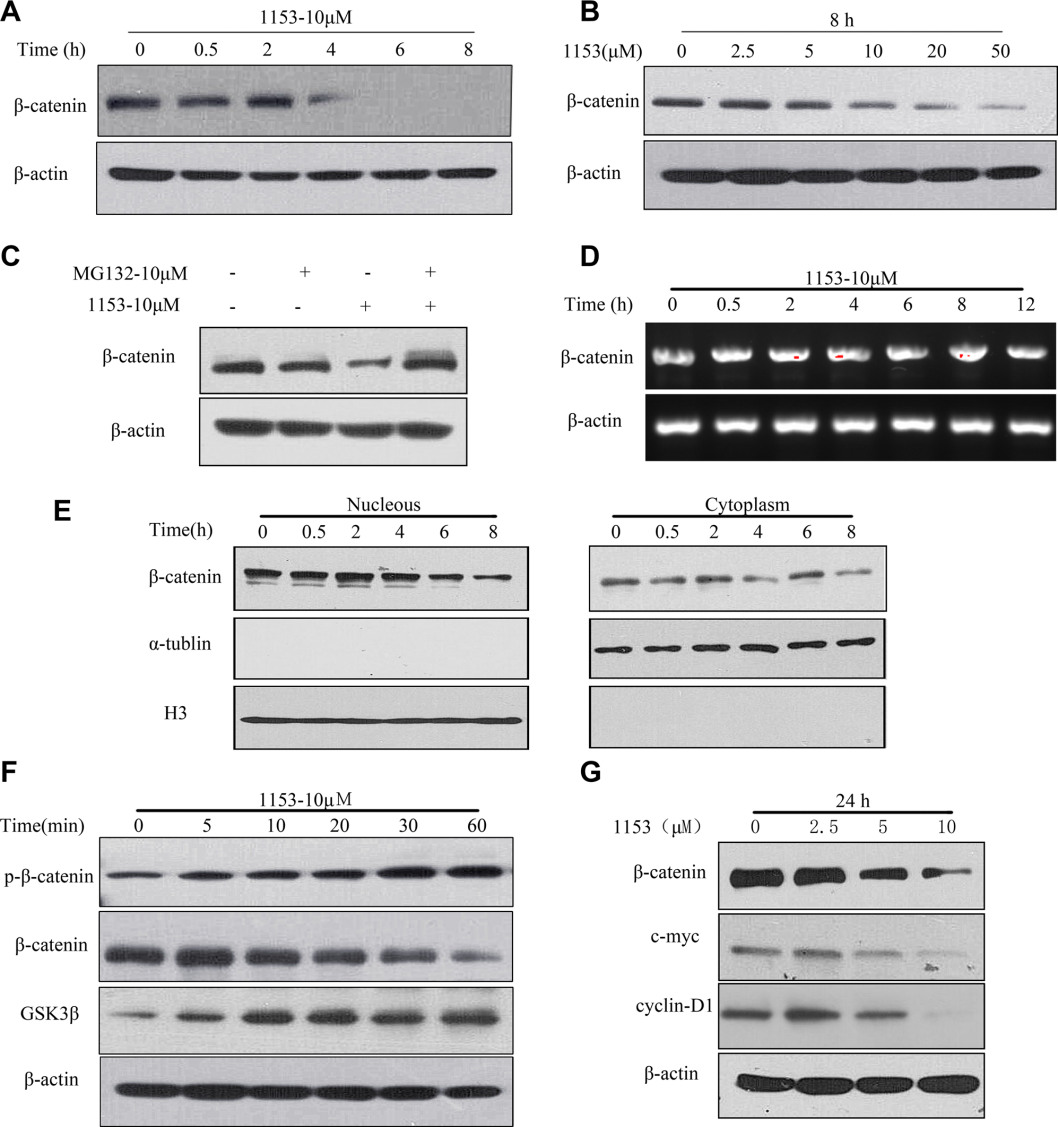
SNG1153 down regulates β-catenin protein level Western blot analysis of the β-catenin expression in H460 tumorsphere cells treated with SNG1153 under the different concentrations of (**A**) or different time periods (**B**). (**C**) Western blot analysis of β-catenin protein level in H460 tumorsphere cells treated with the proteasome inhibitor MG132 and SNG1153 for 2 h. (**D**) RT-PCR analysis of β-catenin mRNA level in H460 tumorsphere cells treated with SNG1153. (**E**) Nucleocytoplasmic separation assay of the β-catenin expression in the nucleus (left) and the cytoplasm (right) in H460 tumorsphere cells treated with SNG1153. (**F**) Western blot analysis of the β-catenin phosphorylation and GSK-3β expression in H460 tumorsphere treated with SNG1153. (**G**) Western blot analysis of the β-catenin downstream genes (c-myc and cyclin-D1) expression in the cells treated with SNG1153. All data are representative results from three independent experiments. 1153: SNG1153.

In the absence of Wnt ligand, β-catenin is phosphorylated at residues Ser45, Thr41, Ser37, and Ser33 by GSK3β, ubiquitinated by the E3 ligase β-TrCP and degraded through the 26S proteasome system. Wnt stimulation leads to the inhibition of β-catenin phosphorylation and degradation, and then β-catenin will translocate and accumulate in the nucleus where it activates the expression of the target genes, such as c-myc and CyclinD1 [28, 45, 46]. We found SNG1153 significantly reduced β-catenin protein level in a time and dose-dependent manner (Figure 5A, 5B), which was reversed by MG132, a proteasome inhibitor (Figure 5C). RT-PCR assay confirmed that β-catenin mRNA level was unchanged in the presence of SNG1153 (Figure 5D). Further investigation by nucleocytoplasmic separation assay showed that SNG1153 reduced β-catenin protein level in tumorsphere cells both in the nucleus and the cytoplasm (Figure 5E). We also found SNG1153 increased GSK 3β expression and induced the β-catenin phosphorylation (Figure 5F). Our results indicated that SNG1153 destabilizes β-catenin protein, and reduces the protein levels of β-catenin presumably through induction of GSK3β expression. As SNG1153 is a synthetic compound derived from icaritin, we next want to study whether they choose different signaling pathways for cancer therapy. Unlike SNG1153, icaritin did not decrease the protein levels of β-catenin, however, SNG153 suppressed Stat3 phosphorylation as icaritin did (Figure S3). These results explained why SNG1153 exerts more potent anticancer activity than icaritin *in vitro*.

To test the effect of SNG1153 on the expression of β-catenin downstream genes, we examined the expression of c-myc and cyclinD1 in H460 tumorspheres treated with SNG1153. We found that SNG1153 down regulated c-myc and cyclinD1 expression in a dose-dependent manner (Figure 5G).

### β-catenin plays a critical role for tumorsphere formation

**Figure 6:**
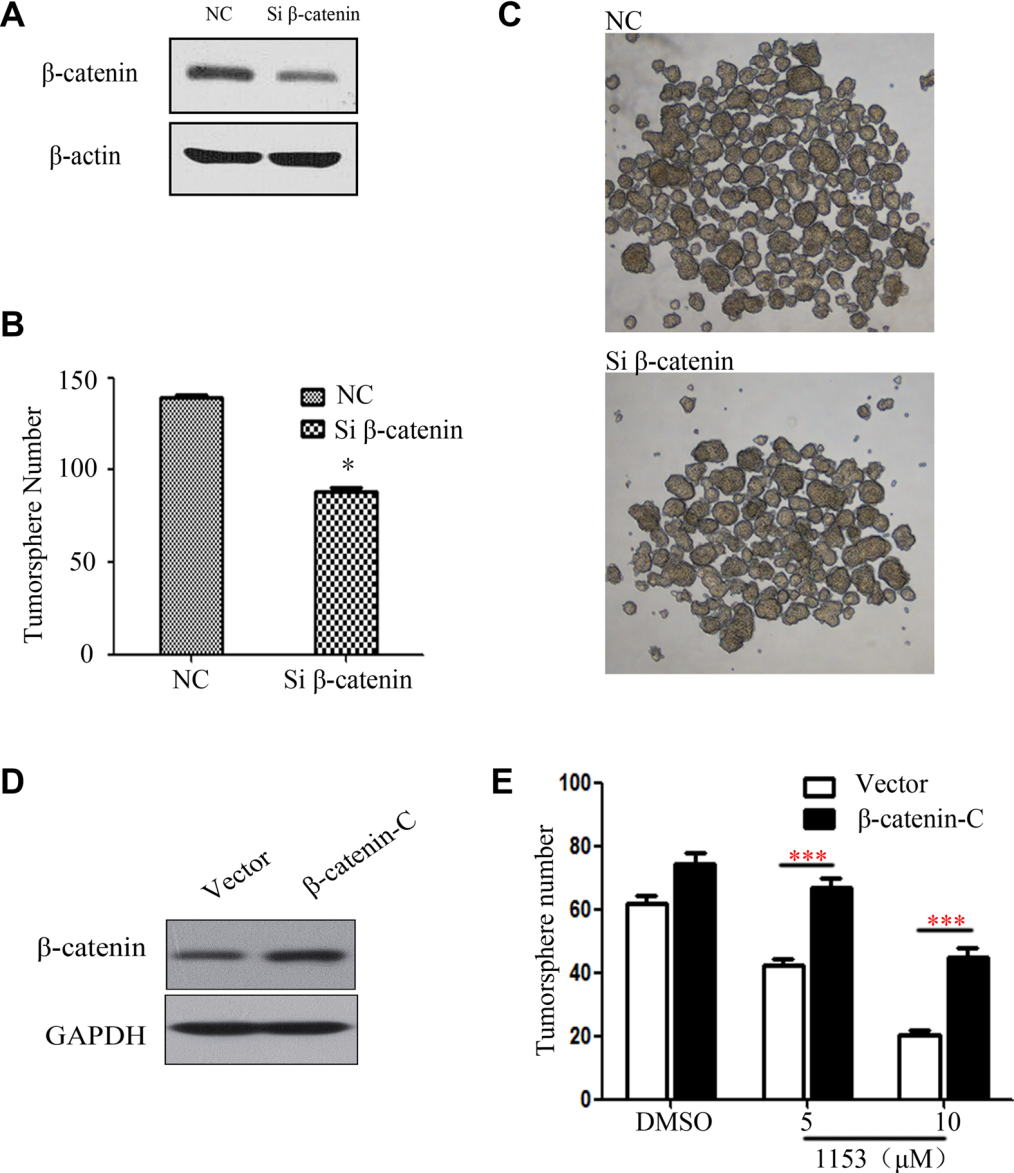
β-catenin Knock down attenuates tumorsphere formation and overexpression of active β-catenin weakens the SNG1153's inhibitory effect on H460 tumorspher. H460 cells transfected with the control siRNA or the β-catenin siRNA for 48 h (**A**) Total cell lysates were prepared and western blots were carried out with the indicated antibodies. (**B** and **C**) H460 were transiently transfected then cultured in tumorsphere medium for 5 days, photographed and counted. (**D** and **E**) H460 cells were transiently transfected with the vector control or the β-catenin-C (β-catenin constitutive active) plasmid for 48 h. Cells were harvested for western blots and tumorsphere formation assay. Data are representative results from three independent experiments. Points, mean (*n* = 3); bars, SEM. *P < 0.05.

To further study the importance of β-catenin in lung cancer stem cells, we used siRNA specific for β-catenin to knock down β-catenin in H460 cells. We found that β-catenin knock down in H460 cells reduced tumorsphere formation (Figure 6A–6C). Thus, β-catenin plays an important role in stemness of tumorsphere cells derived from H460 cells.

### SNG1153 suppresses lung CSC via β-catenin pathway

We next over expressed constitutively active β-catenin in H460 cells to further study whether SNG1153 inhibits lung CSC via β-catenin pathway. Western blot analysis showed that the constitutively active β-catenin (β-catenin-C) increased the expression levels of β-catenin (Figure 6D). Forced expression of β-catenin reduced SNG1153-inhibitory activity in tumorsphere formation (Figure 6E).

## DISCUSSION

SNG1153 is a derivate from icaritin, a hydrolytic product of icariin from traditional Chinese herbal medicine *Epimedium*. The marked difference between SNG1153 and icaritin is that the methyl group at C-4′ in icaritin is replaced by trifluoromethyl. Previous studies showed that icaritin inhibits breast, leukemia, endometrial and liver cancer cell growth [34–38]. Here, we examined the effect of SNG1153 on growth of lung cancer H460 cells. Our data showed SNG1153 has better bioavailability and inhibitory activity than icaritin.

Increasing pieces of evidence indicated that human cancers are driven and sustained by a small population of cells called cancer stem cells [3]. Currently, there are few effective compounds that are targeting cancer stem cells [3, 47, 48]. Many efforts have been concentrated to the identification and characterization of specific inhibitors of CSC homeostasis [49–53]. Curcumin [54, 55] and sulforaphane [56] were reported to inhibit CSC growth. In this study, we demonstrated that SNG1153 inhibited growth of tumorsphere cells derived from human lung cancer H460 cells. We found that SNG1153 inhibited tumorsphere formation and self-renewal. In lung cancer, CD133 is the most useful marker for cancer stem cells, and we found that SNG1153 treatment decreased population of cells positive for CD133. Furthermore, we demonstrated that SNG1153 pretreatment reduced CSC in H460 cells and thus blocked tumor formation *in vivo.* Our results strongly indicated that SNG1153 can inhibit the growth of lung cancer and lung CSCs.

A number of studies have demonstrated that the Wnt/β-catenin pathway is crucial in the maintenance of cancer stem cells in leukemia [57–59], melanoma [60], colon cancer [61] and so on. In the absence of Wnt ligands, the β-catenin is phosphorylated at residues Ser45, Thr41, Ser37, and Ser33 by a multi-protein destruction complex including GSK3β, ubiquitinated by the E3 ligase β-TrCP and then subsequently degraded through the 26S proteasome system. Wnt stimulation leads to the inhibition of β-catenin phosphorylation and degradation, and then β-catenin translocates into the nucleus where it forms a complex with (TCF/LEF) transcription factors, activating its target genes expression, such as c-myc and cyclinD1 [38, 39]. We found SNG1153 treatment reduced the protein levels of β-catenin, which was reversed by MG132. SNG1153 phosphorylated β-catenin and increased GSK3β expression. We also demonstrated SNG1153 suppresses the expression of β-catenin downstream genes, such as c-myc and cyclinD1. Lung CSC inhibition by SNG1153 is β-catenin dependent.

In this study, SNG1153 exhibited anti-growth activity in lung cancer H460 cells, and in tumorsphere cells from H460. SNG1153 inhibits lung cancer cells by down-regulating Wnt/β-catenin signaling pathway. Our results provide a new piece of evidence to support our hypothesis that SNG1153 could be developed into a novel therapeutic agent for treatment of human lung cancer.

## MATERIALS AND METHODS

### Reagents

SNG1153 with a purity of up to 99.5% was obtained from Shenogen Pharma Group, Beijing, China. A stock solution of SNG1153 (50 mM) was prepared with DMSO (Sigma, St Louis, MO, USA). Other chemicals were purchased from Sigma unless otherwise indicated.

### Cell culture and cell lines

The human lung cancer cell line H460 was obtained from the Institute of Basic Medical Sciences, Chinese Academy of Medical Sciences. Cells were maintained in RPMI-1640 medium (Gibco, USA) containing 10% fetal bovine serum (FBS) at 37°C in a humidified atmosphere of 5% CO_2_.

### CCK-8 assay

Cell viability was measured using the CCK-8 assay kit (Dojindo), according to the manufacturer's instruction. Briefly, H460 cells were seeded in 96-well plates to a final density of 3 × 10^3^ /well, and treated with vehicle (DMSO) and different concentrations of the indicated compounds for forty-eight hours. CCK-8 was then added into each well for about 1 h, and the plates were read at wavelength of 450 nm. Three wells were used for each treatment, and the experiments were repeated more than three times. The percentage of viable cells was calculated using the formula: ratio (%) = [OD (Treatment) – OD (Blank)]/[OD (Control) – OD (Blank)] × 100.

### Colony formation assay

For clonogenic assay, the cells were treated with indicated concentrations of SNG1153 or vehicle (DMSO) respectively. After 24 h, the treated cells were suspended in 1 ml medium containing 0.3% low-melting-point agarose (Amresco, Solon, OH, USA), and plated on a bottom layer containing 0.6% agarose in 35 mm plates (1000 cells/plate). After 10 days of culture, cells were stained with Giemsa and colonies containing more than 50 cells were counted and photographed.

### Cell apoptosis assay

H460 cells (2 × 10^5^/well) in six-well plates were treated with different concentrations of SNG1153 or vehicle (DMSO) respectively for 48 h, then collected and washed twice in ice-cold PBS. Cell apoptosis assay was conducted using an AnnexinV-FITC kit (eBioscience, San Diego, CA), according to the manufacturer's instruction and the results were examined with BD LSR II™ flow cytometer (Becton-Dickinson, CA, U.S.) and analyzed with Flow Jo software (Becton-Dickinson, CA, U.S.).

### Tumorsphere culture

H460 cells were suspended in serum-free DMEM/F12 medium (Gibco) containing B27 (Invitrogen), human recombinant epidermal growth factor EGF 20 ng/ml (PeproTech), basic fibroblastic growth factor bFGF 20 ng/ml (PeproTech) and plated at 500 to 30,000 cell/ml in ultralow-attachment 24- or 6-well plates (Corning, USA). The medium was replaced twice a week. Self-renewal capacity of the CSCs was examined by re-populating tumorspheres. Briefly, tumorspheres were collected by centrifugation, trypsinized, passed through a 45 mm strainer (BD, Biosciences), counted and replated in tumorsphere culture medium. Tumorspheres were defined as spheres with a diameter > 100 μm size. The numbers of tumorspheres were quantitated using Image J software (NIH, USA).

### Western blot analysis

The cells were collected and washed with ice-cold PBS and the cell extracts were prepared in RIPA buffer with proteinase inhibitor cocktail (Roche, Germany) and phosphorylation inhibitors (Sigma). Equal amounts of protein were separated by a 10% SDS-PAGE and electro-transferred to a PVDF membrane (Millipore, Temecula, CA). The membranes were then blocked, incubated with primary antibodies overnight at 4°C, followed by incubation with the appropriate secondary antibodies. The membranes were then visualized using the enhanced chemiluminescence detection system (Engreen, China).

Monoclonal antibodies for β-catenin, p-β-catenin, GSK3β, Stat3, P-Stat3 were purchased from Cell Signaling Technology. Anti-cyclinD1 antibody was from Abcam. Antibodies for c-myc, β-actin, Lamin-B, α-tublin, GAPDH and H3 were from Santa Cruz Bio Technology (Santa Cruz, CA. USA).

### FACS assay

H460 were seeded in low attachment plates (Corning) with the concentration of 10,000 cells/ml. Cells were cultured for 5 days, then digested and collected. The cells were re-suspended in PBS at 2 × 10^6^/100 ul. Unstained cells, isotype and single color-stained cells were used for controls. CD133-PE antibody (Miltenyi, Biotec) was used for CD133 staining. The results were examined with BD Accuri™ C6 flow cytometer (Becton-Dickinson, CA, U.S.) and analyzed with CFlow Plus software (Becton-Dickinson, CA, U.S.).

### Animal experiments

All the experimental procedures were approved by the Animal Care and Use Committee of Institute of Chinese Materia Medica, China Academy of Chinese Medical Sciences (Beijing, China). Male, 6–8 weeks old NOD/SCID mice were used (Vitalriver, Beijing, China) in mice animal experiments.

For the tumor seeding ability assay, the bulk and enriched cancer stem cells from H460 lung cancer cells were used. The cells treated with different compounds for 48 h, and then subcutaneously injected into mice at doses of 5 × 10^2^ and 5 × 10^4^ per injection site. Tumor nodules usually became palpable within 2∼4 weeks after cell injection. The length and width of the tumors were measured every day. Tumor volumes were calculated as: tumor volume = (length × width^2^)/2.

### Plasmids and siRNAs transfection

Constitutively active β-catenin plasmid was from Addgene and β-catenin siRNA was from Santa Cruz Bio Technology. H460 cells (2 × 10^5^/well) in a six-well plate were transfected with the indicated plasmids (0.1 μg/ml) or siRNAs (50 nM) by Lipofectamine 3000™ following the manufacturer's instructions (Invitrogen). After 48 h, cells were harvested for the tumorsphere formation assay or western blots.

### RNA extraction and reverse transcription polymerase chain reaction (RT-PCR)

The tumorsphere were seeded at 2 × 10^4^ per well in ultralow-attachment 6-well plates with tumorsphere culture medium in the presence of the indicated agents. RNA was isolated with Trizol (Invitrogen) and cDNA was synthesized according to the manufactory's instruction (AMV First Strand cDNA Synthesis Kit, NEB). RT-PCR was performed in a total reaction volume of 20 μl. The primers for β-catenin and β-actin were as follows: β-catenin, 5′-GAAACGGCTTTCAGTTGAGC-3′ and 5′-TTCCAT CATGGGGTCCATAC-3′; β-actin, 5′-TTCCTTCCTGG GCATGGAGTCCTG-3′and 5′-GAGGAGCAATGATC TTGATCT-3′.

### Statistical analysis

For reach *in vitro* experiment, a minimum of three wells or dishes was used and similar results were usually obtained. Each experiment was repeated at least three times. The values are represented as mean ± SD or SEM as indicated. The difference between groups was analyzed using the Student's *t*-test when comparing only two groups or by a one-way ANOVA analysis when comparing more than two groups. Differences were considered statistically significant at *P < 0.05, **P < 0.01 and ***P < 0.001.

## Supplementary Materials and Figures



## Abbreviations

CSCs: cancer stem cells
RT-PCR: reverse transcription polymerase chain reaction
Stat3: Signal transducer and activator of transcription 3
Oct4: octamer-binding transcription factor 4
GSK3β: glycogen synthase kinase 3 beta
NOD/SCID: non-obese diabetic/severe combined immunodeficient.
