# Supplementary material for: A novel anticancer agent SNG1153 inhibits growth of lung cancer stem/progenitor cells
Source: Oncotarget. 2016 Jun 2;7(29):45158–70. doi: 10.18632/oncotarget.9783 (PMC5216713; doi:10.18632/oncotarget.9783)
Supplement: Supplementary file 1 [file oncotarget-07-45158-s001.pdf]

A novel anticancer agent SNG1153 inhibits growth of lung cancer stem/progenitor cells

Supplementary Materials

Supplementary Table S1: Tumor seeding ability with transplantation from drugs-treated sphere cells

	5×10^2					5×10^4				
**Tumor formation**	**DMSO**	**Taxol 5 nM**	**Taxol 10 nM**	**1153 5 µM**	**1153 10 µM**	**DMSO**	**Taxol 5 nM**	**Taxol 10 nM**	**1153 5 µM**	**1153 10 µM**
n = 6	6/6	6/6	6/6	0/6	0/6	6/6	6/6	6/6	6/6	6/6

Tumorspheres survived from drugs treatment were subcutaneously injected with 5×10^2 and 5×10^4 respectively into each of NOD/SCID mice. Tumor incidence was analyzed after 30 days of cells injection.

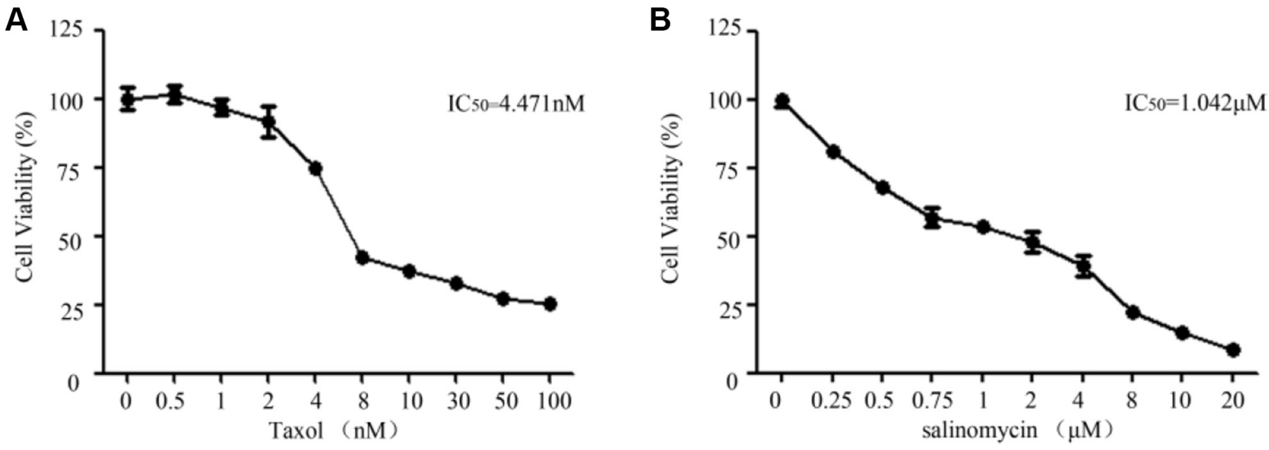

Supplementary Figure S1: Taxol (**A**) and salinomycin (**B**) inhibited H460 cell growth.

A

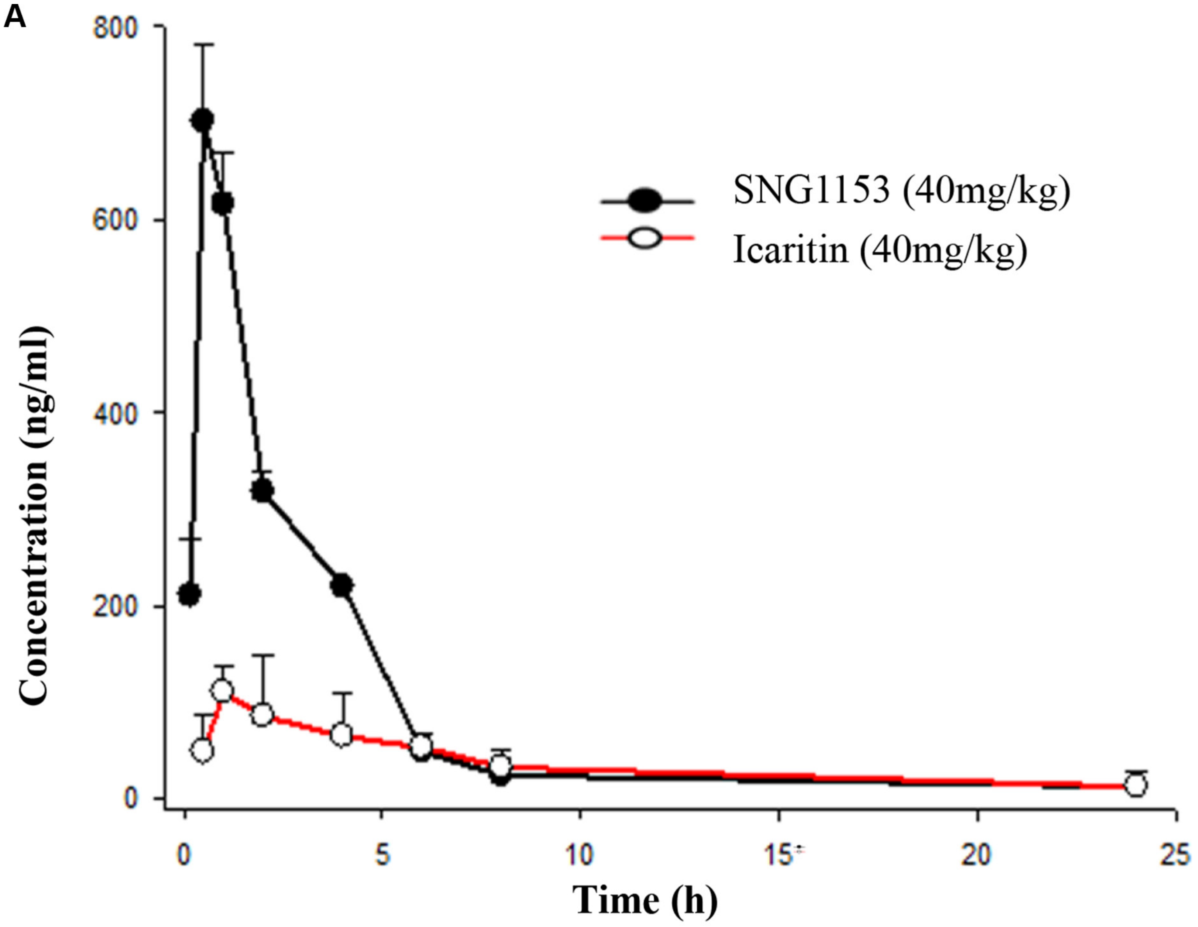

B

H460 IC_{50} (μM)	SNG1153	Icaritin
Mean	1.248	7.911
SD	0.087	0.1905

Supplementary Figure S2: (**A**) The mean plasma concentration-time profiles of SNG1153 and icaritin after oral administration to dogs are shown. Cmax and AUC (area under concentration-time curve) of SNG1153 were significantly higher than icaritin in the plasma. SNG1153 (40 mg/kg) and icaritin (40 mg/kg) were administration via oral gavage to beagle dogs. Blood samples were collected from peripheral veins at 0.5, 1, 2, 4, 6, 8, 24 h after administration. Plasma was obtained after blood samples centrifuged at 2000 g for 10 min, subsequently incubated with Phosphate buffer (pH5.0) containing β-glucuronidase (20000 U/mL) for 1 h at 37, then added with acetonitrile which contains IS (Dexamethasone 50 ng/mL) for protein precipitation. An aliquot of 200 ul of supernatant was injected into the LC-MS/MS system for chemicals concentration analysis (**B**) H460 IC_{50} values of SNG1153 and icaritin.

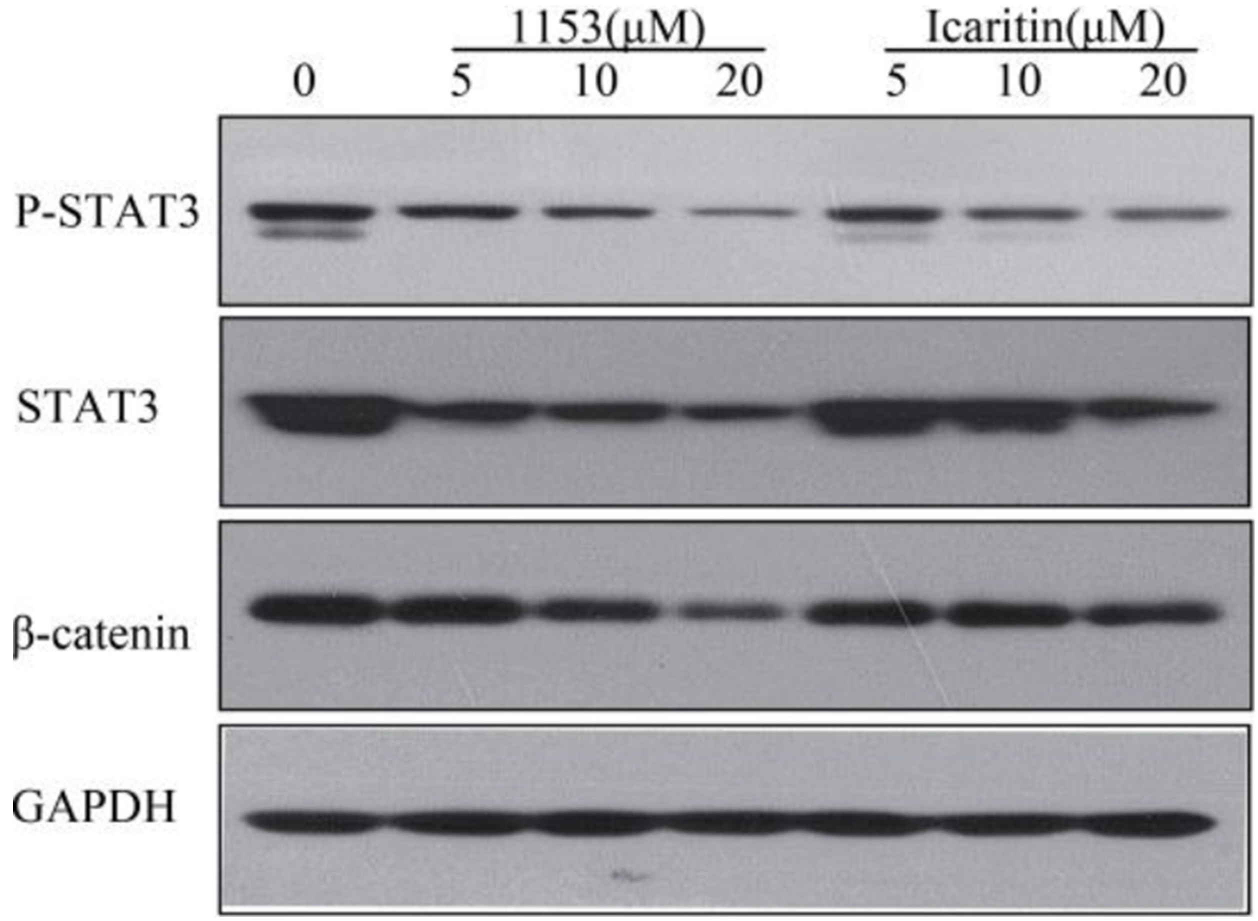

Supplementary Figure S3: Western blot data of the p-Stat3 level and β-catenin expression in H460 tumorsphere cells treated with the different concentrations of SNG1153 and icaritin was shown. GAPDH was used as loading control. 1153, SNG1153.
